# Supplementary material for: Sub-nanowatt resolution direct calorimetry for probing real-time metabolic activity of individual C. elegans worms
Source: Nat Commun. 2020 Jun 12;11:2983. doi: 10.1038/s41467-020-16690-y (PMC7293274; doi:10.1038/s41467-020-16690-y)
Supplement: Supplementary file 1 — Reporting Summary [file 41467_2020_16690_MOESM1_ESM.pdf]

nature research

Corresponding author(s): Swathi Yadlapalli
Pramod Reddy
Edgar Meyhofer

Last updated by author(s): May 8, 2020

Reporting Summary

Nature Research wishes to improve the reproducibility of the work that we publish. This form provides structure for consistency and transparency in reporting. For further information on Nature Research policies, see Authors & Referees and the Editorial Policy Checklist .

Statistics

For all statistical analyses, confirm that the following items are present in the figure legend, table legend, main text, or Methods section.

n/a	Confirmed	
☐	☒	The exact sample size (n) for each experimental group/condition, given as a discrete number and unit of measurement
☐	☒	A statement on whether measurements were taken from distinct samples or whether the same sample was measured repeatedly
☐	☒	The statistical test(s) used AND whether they are one- or two-sided<br>*Only common tests should be described solely by name; describe more complex techniques in the Methods section.*
☐	☒	A description of all covariates tested
☒	☐	A description of any assumptions or corrections, such as tests of normality and adjustment for multiple comparisons
☐	☒	A full description of the statistical parameters including central tendency (e.g. means) or other basic estimates (e.g. regression coefficient) AND variation (e.g. standard deviation) or associated estimates of uncertainty (e.g. confidence intervals)
☐	☒	For null hypothesis testing, the test statistic (e.g. F, t, r) with confidence intervals, effect sizes, degrees of freedom and P value noted<br>*Give P values as exact values whenever suitable.*
☒	☐	For Bayesian analysis, information on the choice of priors and Markov chain Monte Carlo settings
☒	☐	For hierarchical and complex designs, identification of the appropriate level for tests and full reporting of outcomes
☒	☐	Estimates of effect sizes (e.g. Cohen's d, Pearson's r), indicating how they were calculated

Our web collection on statistics for biologists contains articles on many of the points above.

Software and code

Policy information about availability of computer code

Data collection	Raw data were collected with LabView 2018 and Micro-Manager 1.4.22
Data analysis	Data analysis was performed in LabView 2018, Excel 2016, ImageJ 1.51 and Origin 2019b.

For manuscripts utilizing custom algorithms or software that are central to the research but not yet described in published literature, software must be made available to editors/reviewers. We strongly encourage code deposition in a community repository (e.g. GitHub). See the Nature Research guidelines for submitting code & software for further information.

Data

Policy information about availability of data

All manuscripts must include a data availability statement. This statement should provide the following information, where applicable:

- Accession codes, unique identifiers, or web links for publicly available datasets
- A list of figures that have associated raw data
- A description of any restrictions on data availability

Data are available from the corresponding authors upon reasonable request.

Field-specific reporting

Please select the one below that is the best fit for your research. If you are not sure, read the appropriate sections before making your selection.

☒ Life sciences ☐ Behavioural & social sciences ☐ Ecological, evolutionary & environmental sciences

For a reference copy of the document with all sections, see nature.com/documents/nr-reporting-summary-flat.pdf

Life sciences study design

All studies must disclose on these points even when the disclosure is negative.

Sample size	Samples sizes were not pre-determined. We repeated measurements to obtain sample sizes comparable to those used in related studies (BMC Biol 15, 30 (2017): n = 4-10; Lab Chip, 2018,18, 1641-1651: n=3). Previous metabolic studies required measurements averaged over hundreds of samples to resolve and were repeated a few times (comparable to the n stated above) repeats. With our instrument's improved sensitivity, we were able to obtain resolve metabolic outputs of individual worms in independent trials and for age-dependant data with n = 3-14.
Data exclusions	No data were excluded.
Replication	All experiments were repeated multiple times with independent samples. Multiple repeated experiments for device characterization involving thermal conductance, time constant, stability tests revealed consistent results. AMR and SMR of a worm at a given stage of life-cycle were achieved by repeating measurements on independent worms, and the corresponding mean and standard deviations reported.
Randomization	All metabolic heat output measurements were performed on worms selected randomly from agar plates. Randomization is not relevant to the experiments on device characterization as the relevant device characteristics are deterministic.
Blinding	Experiments were performed on two strains of C. elegans. Experiments for one strain at various developmental stage were conducted for several weeks before switching to the other strain. The experimental protocol was identical for both strains, data analysis is automated and requires no input from investigators. Blinding was not used.

Reporting for specific materials, systems and methods

We require information from authors about some types of materials, experimental systems and methods used in many studies. Here, indicate whether each material, system or method listed is relevant to your study. If you are not sure if a list item applies to your research, read the appropriate section before selecting a response.

Materials & experimental systems

n/a	Involved in the study	
☒	☐	Antibodies
☒	☐	Eukaryotic cell lines
☒	☐	Palaeontology
☐	☒	Animals and other organisms
☒	☐	Human research participants
☒	☐	Clinical data

Methods

n/a	Involved in the study	
☒	☐	ChIP-seq
☒	☐	Flow cytometry
☒	☐	MRI-based neuroimaging

Animals and other organisms

Policy information about studies involving animals; ARRIVE guidelines recommended for reporting animal research

Laboratory animals	C. elegans: Bristol N2 (wild-type) and daf-2 (el370). These strain are available at Caenorhabditis Genetics Center (CGC) . We did not distinguish the sex of the worms (hermaphrodite or male). Worms from L1 to adults were used in the study.
Wild animals	This study did not involve wild animals.
Field-collected samples	This study did not involve samples collected from the field.
Ethics oversight	No ethical approval or guidance was required .

Note that full information on the approval of the study protocol must also be provided in the manuscript.
