# Supplementary material for: Sub-nanowatt resolution direct calorimetry for probing real-time metabolic activity of individual C. elegans worms
Source: Nat Commun. 2020 Jun 12;11:2983. doi: 10.1038/s41467-020-16690-y (PMC7293274; doi:10.1038/s41467-020-16690-y)
Supplement: Supplementary file 2 — Supplementary Information [file 41467_2020_16690_MOESM2_ESM.pdf]

Supplementary Information

Sub-nanowatt resolution direct calorimetry for probing real time metabolic activity of individual *C. elegans worms*

Sunghoon Hur†[,1], *Rohith Mittapally*†[,1], *Swathi Yadlapalli*[*,2], *Pramod Reddy*[*,1,3]

and Edgar Meyhofer[*,1,4]

[1]Department of Mechanical Engineering, University of Michigan, Ann Arbor, MI 48109, USA

[2]Department of Cell and Developmental Biology, University of Michigan, Ann Arbor, MI 48109, USA

[3]Department of Materials Science and Engineering, University of Michigan, Ann Arbor, MI 48109, USA

[4]Department of Biomedical Engineering, University of Michigan, Ann Arbor, MI 48109, USA

†These authors contributed equally to this paper.

[*]email: swathi@umich.edu, pramodr@umich.edu, meyhofer@umich.edu

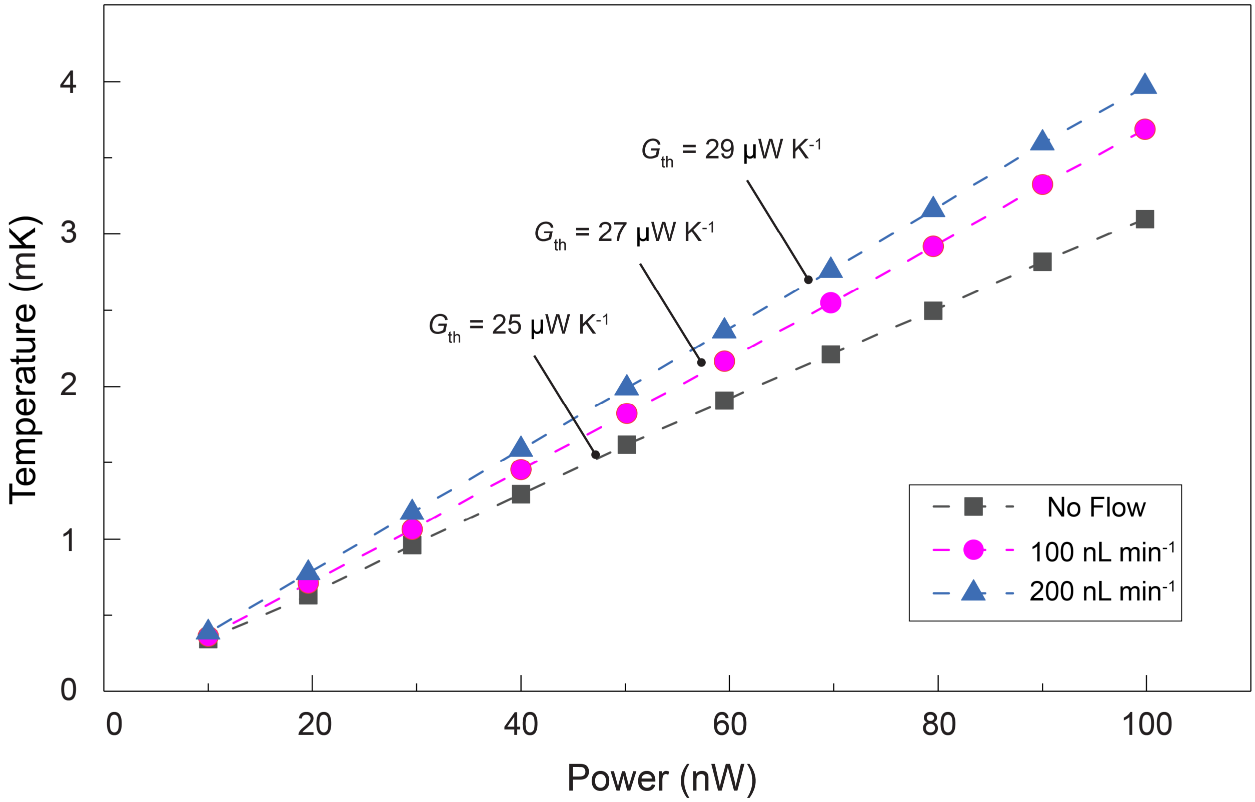

Supplementary Figure 1 | Thermal conductance (G_{th}) measurement under different operation conditions. The thermal conductance, G_{th} , increases as flow rate increases. Our metabolic heat output measurements were performed at a flow rate of 100 nL min^{-1}, which corresponds to a thermal conductance of 27 μW K^{-1}.

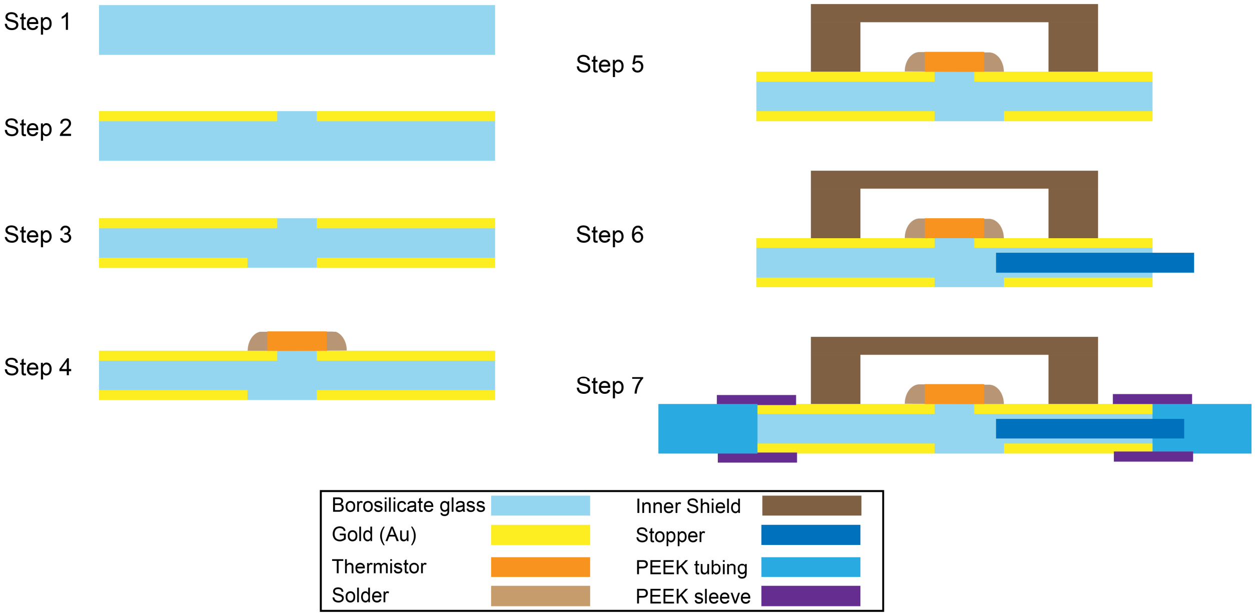

Supplementary Figure 2 | Fabrication steps for the calorimeter. (Step 1) Borosilicate capillary tubes are prepared by chemical and oxygen plasma cleaning. (Step 2) Using a shadow mask, we deposit 10/100 nm Ti/Au to form a metal coating. (Step 3) The tubes are flipped and 10/100 nm Ti/Au are deposited to cover most of the surface of the tubes, including the sides, while leaving a small access window at the center for imaging. (Step 4) A thermistor (Murata Electronics, NCP03, 10 kΩ) is positioned and soldered between the Au films deposited in step 3. (Step 5) The tubes are mounted to the Inner Shield using silver epoxy to achieve good thermal contact with the window facing downwards. (Step 6) A stopper is inserted to the inside of the tube to help localize the worms to the recording area. (Step 7) PEEK tubing is connected to the borosilicate capillary tube using PEEK tubing sleeves.

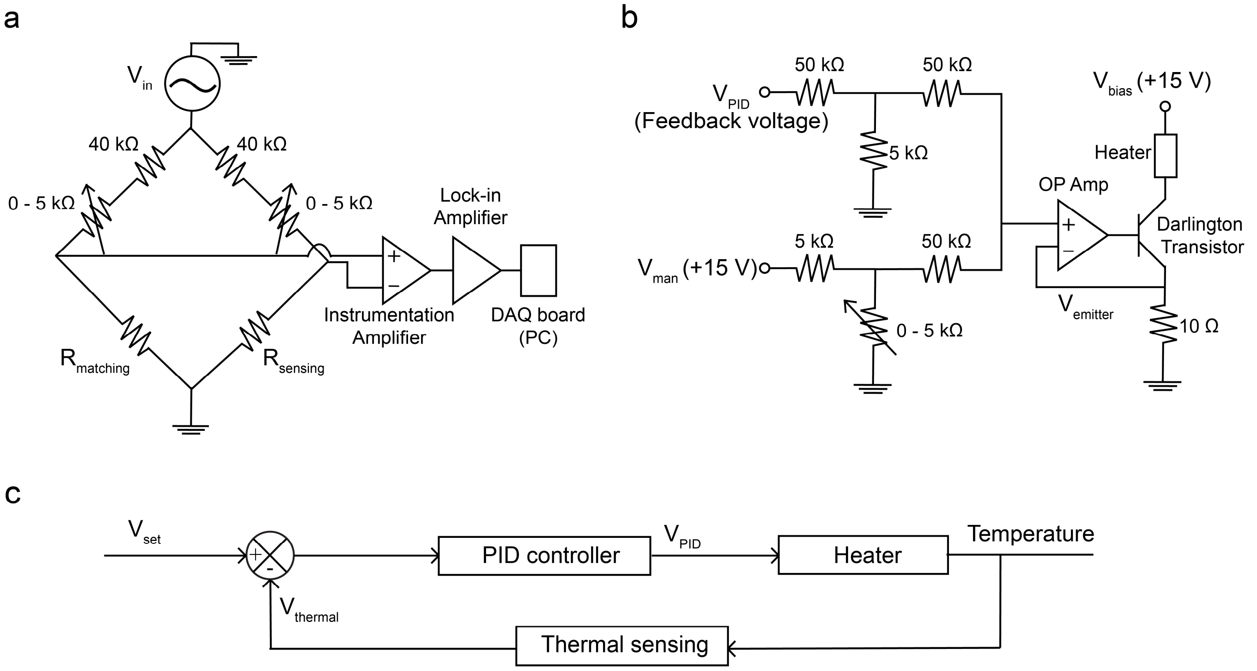

Supplementary Figure 3 | Electronic circuit diagram and schematic of the Proportion Integral Derivative (PID) controller employed for temperature control. **(a)** Circuit diagram for the calorimetric temperature measurement and shields' temperature measurement. When the resistance of the sensing thermistor, $R_{sensing}$, changes due to the heat production by *C. elegans* at the center of the tube, an ac voltage difference, $V_{sensing} – V_{matching}$, develops across the bridge. When the resistance of the sensing thermistor, $R_{sensing}$, changes due to environmental temperature drift, similar changes also arise in the resistance of the matching thermistor $R_{matching}$ and keep the bridge voltage balanced, thus attenuating detrimental effects of temperature drift. The ac output voltage of the bridge circuit is amplified in an instrumentation amplifier (Analog Devices, AD524) and demodulated with a Lock-in Amplifier (Stanford Research Systems, SR830). This output voltage, $V_{thermal}$, is digitized via a data acquisition board (National Instruments, PCI-6014) and a custom LabView (National Instruments) program. We note that similar circuits are used for sensing the shields' temperature by incorporating a precision fixed resistor as $R_{matching}$. **(b)** Schematic of the circuit employed to drive the heater. V_{man} is set to a specific value to supply a constant current to the resistive heater. V_{PID} is added to V_{man} to adjust the current supplied to the heater by controlling the transistor emitter voltage. The current to the heater is set by $V_{emitter}$ divided by 10 Ohm. **(c)** Block diagram of PID feedback loop. Temperature is controlled stably through a PID control system. $V_{thermal}$ is compared to V_{set} and a net voltage is delivered to PID controller. V_{PID} adjusts the

current flow through the resistive heaters (as shown in (b)) and the controlled temperature is read by thermal sensing circuit (as shown in (a)). This loop continues to maintain $V_{thermal}$ to be V_{set}.

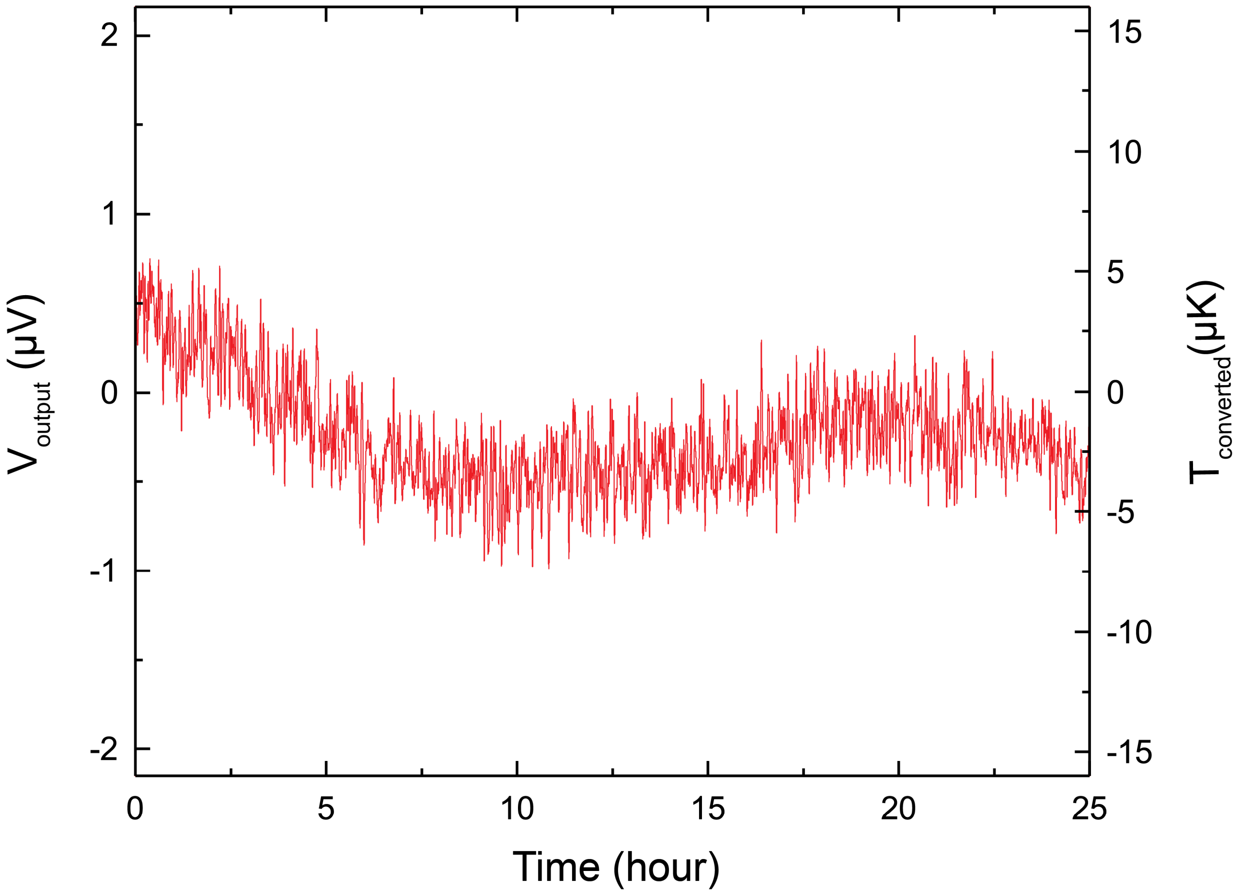

Supplementary Figure 4 | Drift of the output signal of the electronic circuits over a 25 hour period with fixed inputs. For this control measurement the sensing thermistors were replaced with fixed precision resistors. The corresponding temperature drift is ±5 μK.

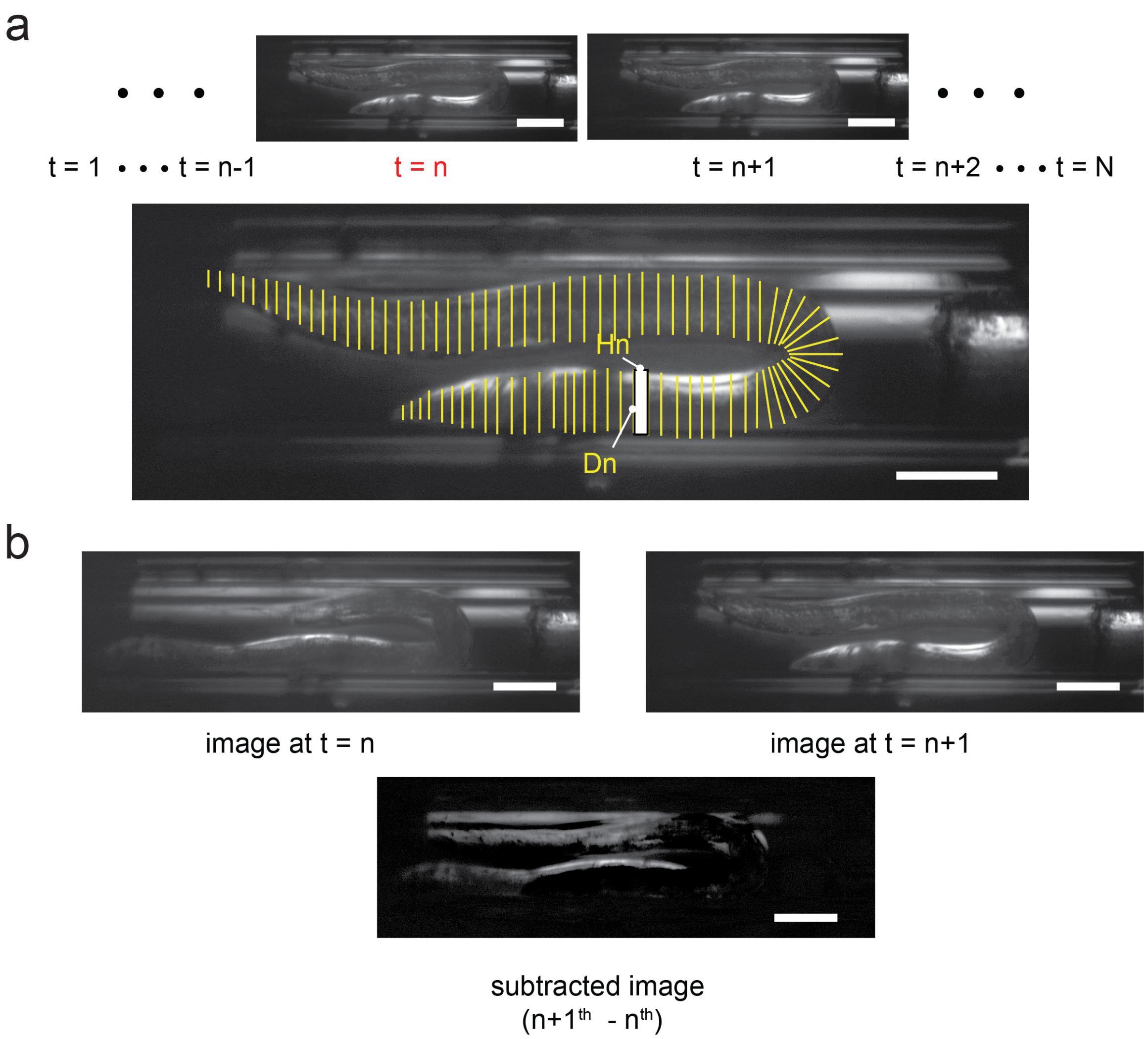

Supplementary Figure 5 | Size measurement and analysis of *C. elegans*. **(a)** *C. elegans* size measurement. (Step 1) Choose a clear image from the acquired image stack. (Step 2) Divide the organism into small sections manually. (Step 3) Sum all volumes by acquiring the diameter (D_n) and width (H_n) of small cylinders. This procedure, applied to independent images (n = 3) in the stack, resulted in similar volumes to within ±5%. The process was repeated for every independent metabolic heat output measurement (n = 61 for N2 wild-type and n = 19 for *daf-2*) **(b)** Activity analysis of *C. elegans*: An image at time t = n, corresponding to the nth image in the stack (top-panel), is subtracted pixel-by-pixel from the $n+1^{th}$ image (middle panel) at time t = n+1, resulting in the subtracted image (bottom panel). The mean intensity (brightness) of all pixels in the subtracted image is calculated to define an activity factor (see Methods section) and indicates the movement of the worm at that point in time during the recording. Scale bars: 100 µm.

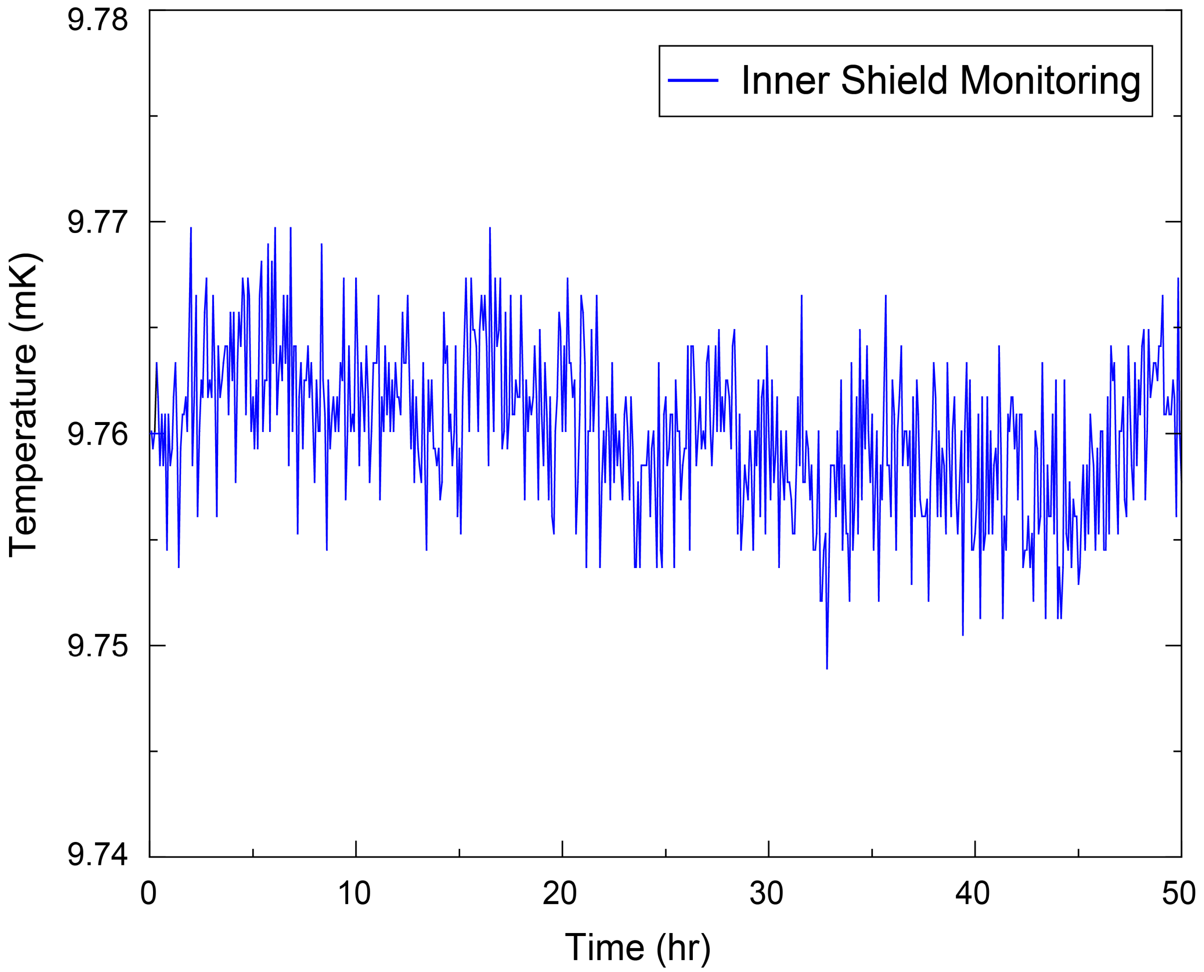

Supplementary Figure 6 | Long-term temperature fluctuations of the inner shield. The monitoring thermistor is affixed on the inner shield on the opposite side relative to the location of the thermistor employed for PID feedback control. Over a period of 2 days, the temperature is stable to within ±15 μK. The drift in the mean value (arbitrary mean of 9.76 mK) over a day is <5 μK.
