# Supplementary material for: Sub-nanowatt resolution direct calorimetry for probing real-time metabolic activity of individual C. elegans worms
Source: Nat Commun. 2020 Jun 12;11:2983. doi: 10.1038/s41467-020-16690-y (PMC7293274; doi:10.1038/s41467-020-16690-y)
Supplement: Supplementary file 4 — Description of Additional Supplementary Files [file 41467_2020_16690_MOESM4_ESM.pdf]

Title: Supplementary Movie 1

Description: Real-time imaging of *C. elegans* for one hour: The top panel shows a video sequence of a worm during a representative measurement of the metabolic heat output in real-time. One second in the video corresponds to one minute in real-time. The middle panel shows the corresponding variation of the calculated activity factor, where the worm's activity is normalized to zero when it is at rest and one at its maximum activity. The third panel shows the temperature change as recorded by our calorimeter. The temperature follows the activity of the worm, but with a lag of few minutes due to the time constant of our device. During the one hour of measurement, the worm alternates between intermittent phases of rest and increased activity.
